# Assessment of Lipocalin 2, Clusterin, Soluble Tumor Necrosis Factor Receptor-1, Interleukin-6, Homocysteine, and Uric Acid Levels in Patients with Psoriasis

**DOI:** 10.1155/2014/541709

**Published:** 2014-04-02

**Authors:** Arzu Ataseven, Recep Kesli, Gulcan Saylam Kurtipek, Perihan Ozturk

**Affiliations:** ^1^Department of Dermatology, Konya Training and Research Hospital, Meram, 42023 Konya, Turkey; ^2^Department of Microbiology, Faculty of Medicine, Afyon Kocatepe University, 03200 Afyon, Turkey; ^3^Department of Dermatology, Faculty of Medicine, Sutcu Imam University, 46050 Kahramanmaras, Turkey

## Abstract

*Background*. Chronic inflammation may play a role in psoriasis pathogenesis. Lipocalin 2, clusterin, soluble tumor necrosis factor receptor-1 (sTNFR-1), interleukin-6, homocysteine, and uric acid are inflammatory and/or biochemical markers. However, both the roles of these markers and the pathogenesis of psoriasis are unknown.* Objective*. The aim of this study was to investigate serum levels of lipocalin 2, clusterin, sTNFR-1, interleukin-6, homocysteine, and uric acid in patients and controls groups.* Methods*. Fifty-six patients with psoriasis and 33 healthy controls were included in the study. Serum concentrations of the markers were evaluated by ELISA. The Psoriasis Area and Severity Index (PASI) was evaluated in all psoriasis patients. Body mass index (BMI) was calculated by dividing weight (kg) by height (m) squared.* Results*. The serum value of lipocalin and sTNFR-1 were significantly higher in psoriasis patients than in controls (resp., *P* < 0.001, *P* < 0.05). The others showed no significant differences between psoriasis and the control groups (all of them *P* > 0.05). The mean PASI score in the patient group was 8.3 ± 6.5.* Conclusions*. These findings suggest that lipocalin 2 and sTNFR-1 might play a role in the pathogenesis of psoriasis and can be used as markers of the disease.

## 1. Introduction

Psoriasis is a chronic recurrent autoimmune skin disease which shows multifactorial etiology and polygenic genetic transmission. It is considered that numerous proinflammatory cytokines, such as TNF-alpha, IL-1, IL-2, IL-6, IL-8, and IL-12, have the main role on the pathogenesis and they are secreted by T cells infiltrated to the skin, in response to unspecified antigenic stimulus. On the other hand, keratinocyte proliferation is a secondary biological phenomenon [1].

Lipocalin 2 (LCN2), also known as neutrophil gelatinase-associated lipocalin, is a 25 kDa protein. LCN2 has been defined as an adipokine. It is expressed in liver, lungs, and kidneys, as well as adipocytes, macrophages, and epithelial cells [2]. LCN2 is an antimicrobial protein [3]. It is stored in specific granules of the human neutrophil and functions as a modulator of inflammation [4]. LCN2 has been demonstrated to be a proinflammatory molecule causing some to call it a cytokine. LCN2 expression is upregulated in various acute and chronic inflammatory diseases such as psoriasis, eczema, periodontitis, and myocarditis [5].

Clusterin (CLU), also known as apolipoprotein J, is encoded on chromosome 8 in humans. It is excreted from various tissues, such as the brain, neural tissue, liver, adrenal glands, and testes. It can be found in serum, cerebrospinal fluid, mother's milk, semen, and urine [6, 7]. CLU has important functions in a number of physiological processes. Moreover, CLU may play a role in numerous processes, including autoimmunity, inflammation, and immunological activities [8].

Soluble tumor necrosis factor receptor-1 (sTNFR-1): tumor necrosis factor (TNF) plays an important role in various events, such as the induction of other cytokines, cell proliferation, and differentiation [9]. There are two receptors for TNF: tumor necrosis factor receptor- (TNFR-) 1 and TNFR-2. TNFR-1 is expressed ubiquitously and is the major receptor. It mediates the effects of soluble TNF-*α* on cells [10]. TNF plays an essential role in the pathogenesis of psoriasis, but its mechanism of action is not fully understood [11].

Interleukin-6 (IL-6) is one of the most important inflammatory cytokines. It is a component of normal human skin and it has been immunologically detected in basal keratinocytes, endothelial cells, mononuclear cells, and fibroblasts [12]. IL-6 plays a central role in a variety of host defense mechanisms, such as the immune response, hematopoiesis, and acute-phase reactions [13]. The human IL-6 gene is located on the short arm of chromosome 7 [14]. IL-6-knockout mice exhibit impaired immune and acute-phase responses [15].

Homocysteineis an *α*-amino acid [16]. It is not obtained from the diet; it is biosynthesized from methionine [17]. A high level of homocysteine increases susceptibility to endothelial injury, which leads to vascular inflammation. This in turn may lead to atherogenesis, which can result in ischemic injury [18]. Hyperhomocysteinemia represents an independent risk factor for atherosclerotic cardiovascular disease [19]. Psoriasis is a systemic, chronic inflammatory skin disease associated with increased cardiovascular risk.

Uric acid (UA) is a product of the metabolic breakdown of purine nucleotides [20].

The aim of this study is to investigate the possible roles of LCN2, an inflammatory adipocytokine; CLU, an enigmatic protein associated with inflammation; sTNFR-1 and IL-6, which are among the proinflammatory cytokines; homocysteine and UA in the pathogenesis of psoriasis, that is, an inflammatory skin disorder.

## 2. Materials and Methods

Fifty-six patients and 33 age- and sex-matched healty controls were prospectively enrolled in this study. Participants gave their informed consent before enrollment. Local ethics committee approval was obtained. Medical history and clinical examinations were performed in both psoriasis and control groups. Clinically and/or histopathologically diagnosed psoriasis was evaluated. PASI scores of the patients with psoriasis were recorded. BMI was calculated as weight/height^2^ (kg/m^2^).

### 2.1. Exclusion Criteria of Patients and Control Groups

Patients who received systemic medication and/or phototherapy were excluded from the study. Other exclusion factors were as follows: BMI ≥ 35, psoriatic arthritis, pregnancy, age < 18 years, smoking, alcohol consumption, hypertension, diabetes mellitus, chronic renal failure, liver and cardiac failure, acute and chronic infections, autoimmune diseases, and cancer.

### 2.2. Laboratory Investigations

Peripheral blood samples were obtained from the study and control groups using blood collector tubes. Serum samples were obtained by centrifugation (3500 rpm, 4 min) and were stored in a deep freezer (−80°C) until use. LCN2, CLU, sTNFR-1, IL-6, homocysteine, and UA levels were measured in serum samples from patients and controls. ELISA kits for LCN2 (LCN2 NGAL/Human ELISA Kit, BioVendor R&D Laboratorni Medicina a.s., Karasek, Czech Republic), CLU (CLU (apolipoprotein J) Human ELISA Kit, BioVendor R&D), sTNFR-1 (Human sTNFR (60 kDa) Platinum ELISA Kit, eBioscience, Bender MedSystems GmbH, Vienna, Austria), IL-6 (Human IL-6 (interferon-beta 2, B-cell stimulatory factor 2, and HGF) Platinum ELISA Kit, eBioscience), and a microplate reader (BioTek ELx 800, BioTek Instrumentations, Inc., Winooski, VT, USA) were used.

Absorbances for all four tests were read at a wavelength of 450 nm. Assay units were ng/mL for LCN2, CLU, and sTNFR, and pg/mL for IL-6. The limit of detection for LCN-2 was 0.02 ng/mL, the standard curve range was 0.3–10 ng/mL, and the calculated overall intra-assay (within-run) coefficient of variation (CV) was 7.7%. The limit of detection for CLU was 0.5 ng/mL, the detection range was 5–160 ng/mL, and the intra-assay CV was 6.2%. The sensitivity for sTNFR-1 was 0.05 ng/mL, the detection range was 0.08–5 ng/mL, and the intra-assay CV was 1.9%. The sensitivity for IL-6 was 0.92 pg/mL, the detection range was 1.56–100 pg/mL, and the intra-assay CV was 3.4%. Six standards and one blank were used for each of the four tests. Homocysteine assays (Homocysteine, Siemens Healthcare Diagnostics Products GmbH, Marburg, Germany) were performed by a chemiluminescence method using an automated immunoassay device (Immulite 2000 XPi Immunoassay System, Siemens Healthcare Diagnostics Inc., Tarrytown, NY, USA), and uric acid assays were performed by a photometric method (Dimension EXL, Siemens Healthcare Diagnostics Inc. Tarrytown, NY, USA) using an automated immunochemistry analyzer manufactured by the same company.

### 2.3. Statistical Analysis

Statistical analysis was performed by SPSS Portable PASW Statistics 18. Student's *t*-test and the chi-squared test were used for group comparisions. Correlations between LCN2, CLU, TNFR-1, IL-6, homocysteine, and UA levels were evaluated by Pearson's correlation test. *P* values < 0.05 were considered to indicate significance.

## 3. Results

The clinical and laboratory characteristics of the patients and control subjects are summarized in Table 1. There were no significant differences between the groups concerning age, sex, or BMI (*P* > 0.05).

**Figure 1:**
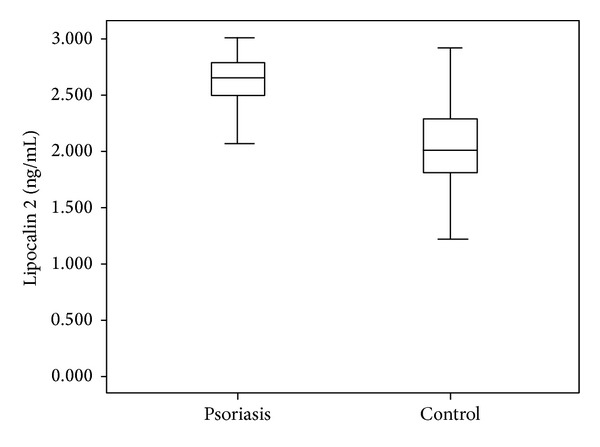
Lipocalin 2 levels in psoriasis and control groups.

**Figure 2:**
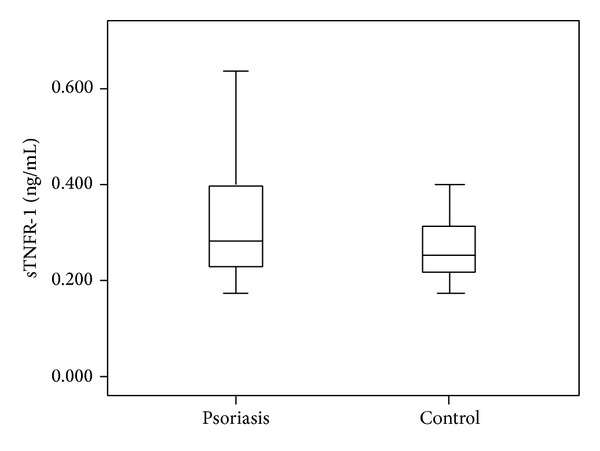
sTNFR-1 levels in psoriasis and control groups.

**Figure 3:**
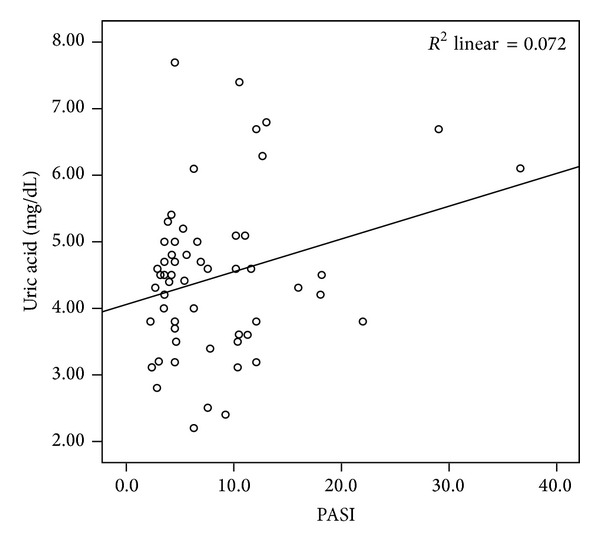
Positive correlation of uric acid levels and PASI.

**Figure 4:**
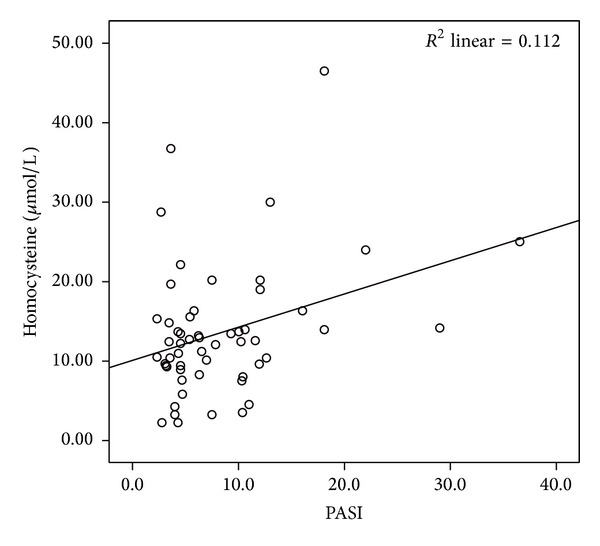
Positive correlation of homocysteine levels and PASI.

**Figure 5:**
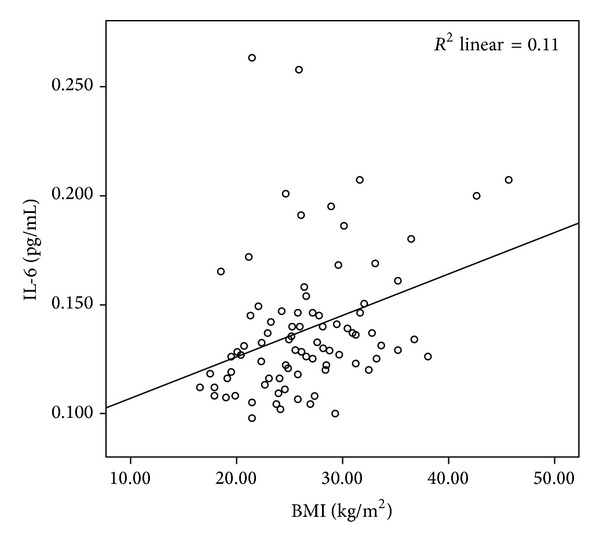
Positive correlation of IL-6 levels and BMI.

**Figure 6:**
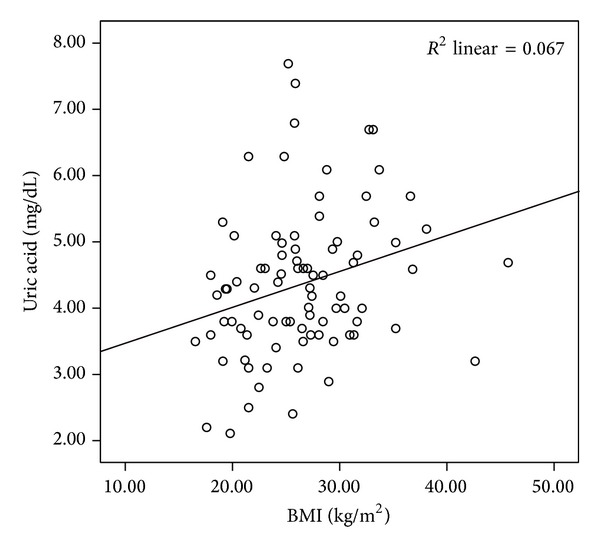
Positive correlation of uric acid levels and BMI.

The mean LCN2 level was 2.54 ± 0.46 ng/mL in the patient group and 2.07 ± 0.39 ng/mL in the control group. The mean CLU level was 2.55 ± 0.26 ng/mL in the patients and 2.52 ± 0.18 ng/mL in the controls. The mean sTNFR-1 level was 0.33 ± 0.15 ng/mL in the patient group and 0.26 ± 0.06 ng/mL in the control group. Comparisons of the serum LCN2 and sTNFR-1 levels of the patient and the control groups revealed statistically significant difference (*P* < 0.001, *P* < 0.05, resp.) (Figures 1 and 2). The mean IL-6 level was 0.14 ± 0.3 pg/mL in patients and 0.13 ± 0.02 pg/mL in the control group. The mean homocysteine level was 13.55 ± 8.3 *μ*mol/mL in patients and 16.04 ± 9.3 *μ*mol/mL in the control group. The mean UA level was 4.48 ± 1.2 mg/dL in patients and 4.20 ± 0.9 mg/dL in the control group. There were no significant differences in CLU, IL-6, homocysteine, or UA levels between psoriasis patients and the control group (*P* > 0.05). The mean PASI score in the patient group was 8.3 ± 6.5. Mean BMI was 26.7 ± 5.8 in patients and 25.7 ± 5.1 in the control group (*P* > 0.05). PASI showed significant positive correlations with homocysteine (*r*: 0.334; *P*: 0.013) and UA (*r*: 0.268; *P*: 0.046) levels (Figures 3 and 4). BMI showed significant positive correlations with UA (*r*: 0.258; *P*: 0.016) and IL-6 (*r*: 0.331; *P*: 0.002) levels (Figures 5 and 6). Other parameters were not correlated with PASI and BMI.

## 4. Discussion

In this study, we found that serum LCN2 levels were significantly increased in patients with psoriasis compared with healty controls. There are a limited number of previous studies of LCN2 in psoriasis. Kamata et al. [2] found that serum LCN2 levels were significantly higher compared to those in healthy controls. Romani et al. [21] also found significantly higher levels of serum LCN2 compared to healthy controls. These two studies were similar to ours and demonstrated high levels of LCN2. However, a study by El-Hadidi et al. [22] did not show significant differences in LCN2 levels compared to controls. Despite this, the authors noted that tissue LCN2 levels were significantly higher [23]. Another study showed that LCN2 gene expression is induced by IL-17 [24]. Lee et al. investigated psoriasis-like diseases and found that in vitro LCN2 expression was highly increased in calcium-induced keratinocyte differentiation [25]. Other researchers also detected increased levels of tissue LCN2 expression using in situ hybridization, RT-PCR, and immunohistochemical methods [26, 27]. Similar to our findings, the previous studies demonstrated that serum and/or tissue levels of LCN2 were generally increased. There are conflicting results regarding the correlation between LCN2 and PASI [2–4, 21]. These differences can be attributed to several factors. One of the main causes is the fact that LCN2 is secreted from various tissues, such as liver, lungs, and kidneys, as well as adipocytes, macrophages, and epithelial cells. Other factors, such as the diversity of patient groups consisting of individuals with different disease durations and severities, past medical history, and medical therapies, may also explain the conflicting results. LCN2 may not completely reflect the degree of skin inflammation of all patients with psoriasis.

Despite LCN2 being an adipokine, we found no significant correlation between LCN2 levels and BMI in either patients with psoriasis or in controls. Kamata et al. [2] also found no relationship between LCN2 and BMI in psoriasis patients and controls. Stejskal et al. [28] also reported no correlation between LCN2 and BMI in patients with metabolic syndrome. However, other studies reported different results [22]. Also in psoriasis, LCN2 has been investigated in metabolic syndrome, atherosclerosis, obesity, and diabetes mellitus [22, 29, 30]. There was no significant difference in the serum CLU levels in psoriasis patients compared to healthy controls in our study. To our knowledge, this is the second study to assess CLU levels in psoriasis patients. The first was conducted in 21 psoriasis patients and 11 healthy controls by Garcia-Rodriguez et al. [31], who found lower plasma CLU levels in psoriasis patients. Recent studies found that CLU has anti-iflammatory properties. The severity of autoimmune myocarditis was increased in CLU-deficient mice, and inflammation progressed rapidly [32]. Similarly, CLU was highly expressed in the synovia of patients with rheumatoid arthritis [33].

The findings concerning CLU levels are contradictory. Its level is increased in some cancers, including prostate, ovary, and bladder cancers, and this increase was associated with a poor prognosis [34]. Fandridis et al. [35] determined that sCLU is involved in both inflammatory and apoptotic molecular processes and noted a high sCLU concentration in human serum and synovial fluid. Wang et al. [36] concluded that decreased CLU levels affect the pathogenesis of SLE due to the diminished protective effects.

Tobisawa et al. [37] retrospectively performed immunostaining in the patients with mycosis fungoides and Sézary syndrome. They claimed that CLU expression could be used as a prognostic marker in early-stage mycosis fungoides to determine the progression to a poor prognosis. Serum CLU levels were found to be higher in patients with systemic sclerosis by Yanaba et al. [38], who noted that the patients with higher CLU levels experienced digital ulcer and pulmonary arterial hypertension less frequently. CLU has a protective role in pulmonary arterial hypertension and digital ulcer. Although the various biological functions of CLU have long been investigated, it remains an enigmatic protein [39]. Hence, additional studies are required to understand the role of CLU as a novel molecule in psoriasis.

In the present study, the soluble TNFR-1 level was higher in psoriasis patients than in controls. There are conflicting results in the literature. Serwin et al. [40] found that serum TNFR-1 levels were significantly increased in patients with psoriasis compared with those in controls. Brotas et al. [41] reported that TNF-*α* has various effects on the cellular level in psoriasis. Coimbra et al. [42] found no differences in TNF levels between patients and controls. Based on the results of this study, we conclude that TNFR-1 level plays a role in the etiopathogenesis of psoriasis.

The serum IL-6 levels did not differ between the psoriasis patients and controls in this study. Increased serum levels of IL-6 have been reported in psoriasis patients [43–45]. As in our study, Kaur et al. [46] reported a statistically significant elevation of IL-6 concentration in obese psoriasis patients. However, in psoriasis patients with normal body weights, the increase was not significant. One possible explanation for the absence of high IL-6 levels in our patients is their lower BMI than those in the study of Kaur et al.

In our study, there were no significant differences in serum homocysteine levels between psoriasis patients and healthy controls. Serum homocysteine levels in patients with psoriasis were positively correlated with PASI. Several researchers have demonstrated significantly increased homocsyteine levels in the psoriasis patients compared with controls [19, 47–52]. However, Cakmak et al. [53] in the Central Anatolia Region of Turkey, the same region as in our study, reported no significant difference in homocysteine levels between psoriasis patients and controls, although serum homocysteine levels correlated with PASI, in agreement with our findings. The difference in findings may be related to regional and/or genetic differences between psoriasis patients. Few studies have shown a relationship between homocysteine levels and PASI [48, 52, 53]. The increased cardiovascular risk with increasing psoriasis severity might be due to homocysteine.

In the present study, serum UA levels did not differ significantly between psoriasis patients and controls. However, serum UA levels were correlated with PASI and BMI. According to previous reports, serum UA levels can be higher in up to 30–50% of psoriasis patients. This can be attributed to the increased epidermal proliferation and associated DNA destruction in psoriasis [54]. Some studies have reported increased UA levels in psoriasis [55–57]. A positive correlation between UA levels and PASI and BMI has been reported [58].

After 12 weeks of therapy, a significant reduction of mean UA level was observed in psoriasis patients. Moreover, increased UA levels were accompanied by increased serum C-reactive protein levels [59]. In agreement with our findings, Kwon et al. [58] did not find high UA levels in patients with psoriasis. High UA levels might be associated with disease severity; another explanation is nutrition-related factors [60], such as obesity and metabolic syndrome. The absence of increased UA levels in our study might be due to the lower disease severity (the mean PASI score was not high) and BMI values of our patients.

In conclusion, LCN2 and sTNFR-1 likely play meaningful roles in the etiopathogenesis of psoriasis. CLU, IL-6, homocysteine, and UA levels did not differ between the patient and healthy control groups. However, in psoriasis patients, the homocysteine and UA levels were correlated with the PASI score, an index of disease severity. The increased cardiovascular risk with increasing psoriasis severity might be caused by homocysteine.

## Figures and Tables

**Table 1:** Demographic and laboratory findings of psoriasis and healthy control groups.

	Psoriasis	Control
*n*	56	33
Age	39.75 ± 18.29	36.18 ± 14.19
Male/female	21/35	11/22
PASI	8.3 ± 6.5	
BMI (kg/m^2^)	26.7 ± 5.8	25.7 ± 5.1
Lipocalin 2 (ng/mL)	2.54 ± 0.46**	**2.07 ± 0.39**
Clusterin (ng/mL)	2.55 ± 0.26	2.52 ± 0.18
sTNFR-1 (ng/mL)	0.33 ± 0.15*	**0.26 ± 0.06**
IL-6 (pg/mL)	0.14 ± 0.3	0.13 ± 0.02
Homocysteine (*μ*mol/L)	13.55 ± 8.3	16.04 ± 9.3
Uric acid (mg/dL)	4.48 ± 1.2	4.20 ± 0.9

PASI: psoriasis area severity index; BMI: body mass index.

*P < 0.05;  **P < 0.001.
